# Sarcopenia Predicts Mortality in Bladder Cancer with Neoadjuvant Chemotherapy: A Multicenter Study

**DOI:** 10.3390/cancers18020222

**Published:** 2026-01-11

**Authors:** Alice Pitout, Charles Mazeaud, Alicia Blondeau, Julia Salleron, Vincent Massard, Aurélien Lambert

**Affiliations:** 1 Department of Urology, CHRU de Nancy, 54500 Vandoeuvre -lès-Nancy, France; 2 Adaptive Diagnostic and Interventional Imaging, INSERM, IADI U1254, Université de Lorraine, 54500 Vandoeuvre-lès-Nancy, France; 3 Biostatistics Unit, Institut de Cancérologie de Lorraine, 54500 Vandoeuvre-lès-Nancy, France; 4 Department of Medical Oncology, Institut de Cancérologie de Lorraine, 54500 Vandoeuvre-lès-Nancy, France; 5 Interdisciplinarity in Public Health, Complex Interventions & Measurement Tools, INSERM INSPIIRE UMR 1319, Université de Loraine, 54500 Vandoeuvre-lès-Nancy, France

**Keywords:** bladder cancer, body composition, chemotherapy, neoadjuvant, sarcopenia

## Abstract

**Background:** Sarcopenia is a recognized adverse prognostic factor in many cancers and can be reliably assessed using computed tomography (CT) scans. Its prognostic value in bladder cancer patients undergoing neoadjuvant chemotherapy remains underexplored. This study aimed to assess sarcopenia’s impact on survival and compare different measurement thresholds. **Methods:** We conducted a retrospective multicenter study including patients with invasive urothelial carcinoma treated with neoadjuvant chemotherapy followed by cystectomy between 2015 and 2021. Sarcopenia was assessed by measuring the Skeletal Muscle Index on CT scans before chemotherapy (BC) and prior to surgery (BS). The primary endpoint was overall survival. Secondary endpoints included progression-free survival (PFS), pathological complete response (pCR), and treatment-related complications. **Results:** Seventy-four patients were included, the majority receiving the MVAC regimen (71.7%). Forty percent of patients achieved a pCR, 35% experienced disease recurrence, and the median PFS was 25 months. Sarcopenia was observed in 27% of patients BC and in 39% BS. Sarcopenia was associated with an increased risk of all-cause mortality: BC according to the definition by Martin et al. (HR 3.38; 95% CI [1.25–9.12]; *p* = 0.016) and Fearon et al. (HR 4.03; 95% CI [1.13–14.3]; *p* = 0.031); and BS according to Martin et al. (HR 3.7; 95% CI [1.12–12.2]; *p* = 0.032) and Fearon et al. (HR 6.08; 95% CI [1.48–24.9]; *p* = 0.012). Sarcopenia was an independent risk factor of shorter PFS. **Conclusions:** Sarcopenia represent an independent and reproducible prognostic factor for mortality in patients with bladder cancer. The study is the first study to compare threshold values at different time points.

## Simple Summary

This study emphasizes the importance of incorporating sarcopenia assessment into the pre-therapeutic evaluation of patients with muscle-invasive bladder cancer treated with neoadjuvant chemotherapy followed by cystectomy. We describe a radiological approach that is simple, rapid, and highly reproducible, based on a semi-automated measurement of skeletal muscle area at the L3 vertebral level on routine CT scans performed before chemotherapy and before surgery. Sarcopenia identified at each stage of treatment was shown to be an independent factor associated with increased overall and specific mortality. These findings were consistent across the two sarcopenia definitions currently validated in the literature, namely those proposed by Martin and Fearon, reinforcing the robustness of our results. These data suggest that sarcopenia represents a clinically relevant biomarker in this population. Early identification of sarcopenic patients should prompt consideration of tailored, preemptive therapeutic strategies, including nutritional and supportive interventions, with the aim of improving oncological outcomes and survival.

## 1. Introduction

Bladder cancer is a prevalent malignancy with a poor prognosis, predominantly affecting men in the latter half of life, with smoking being the principal risk factor. Radical cystectomy remains the standard curative treatment for muscle-invasive disease, but it is associated with complication rates of up to 69% [1], overall mortality of up to 9%, and up to 15% in elderly patients [2,3]. Neoadjuvant cisplatin-based chemotherapy (NAC) is recommended before surgery for eligible patients to improve survival. Its effect is even more pronounced in patients achieving a pathological complete response [4]. Yet, only about half of patients actually receive chemotherapy [5]. Patient selection was based on renal, cardiac, and neurological function. Even when administered, nearly 88% of patients fail to complete the full recommended dose, and 5% discontinue treatment prematurely because of renal toxicity [6].

Early identification of risk factors for treatment failure is therefore critical, as addressing modifiable risks can improve outcomes. Numerous prognostic tools have been explored, including screening questionnaires and biological markers [7]. Nutritional status emerging as a key determinant of prognosis [8]. Over the past two decades, sarcopenia has been recognized as a prognostic factor in multiple cancers [9]. Sarcopenia is characterized by loss of muscle mass and quality. While sarcopenia occurs naturally with aging and is influenced by genetic and lifestyle factors [10], it may be accelerated by acute stressors such as tumor progression. Its severity can be assessed through functional tests, anthropometry, and imaging, with computed tomography (CT) offering a reproducible, accessible, and accurate method [11].

In bladder cancer, research on sarcopenia has been limited and results have been inconsistent, often constrained by small cohort sizes and a focus solely on radical cystectomy [12]. The present study therefore aimed to evaluate whether sarcopenia, assessed using semi-automated CT scans measurements, constitutes a significant prognostic factor for survival and postoperative complications in patients with muscle-invasive bladder cancer treated with neoadjuvant chemotherapy followed by surgery.

## 2. Materials and Methods

We retrospectively collected data from two academic institutions, a University Hospital and a Comprehensive Cancer Care Center, between 2015 and 2021. The study protocol was approved by the French National Commission on Informatics and Liberties (CNIL) and the Research Ethics Board of the Institut de Cancérologie de Lorraine.

### 2.1. Population

The study population consisted of men and women aged over 18 years with urothelial bladder carcinoma who received neoadjuvant cisplatin-based chemotherapy followed by radical cystectomy. In addition, raw data from staging CT scans prior to chemotherapy had to be available.

Patients were eligible if they had received at least one cycle of chemotherapy or had undergone surgery at one of the two participating academic centers. Chemotherapy regimens included cisplatin-gemcitabine (GC) or methotrexate, vinblastine, adriamycin, and cisplatin (MVAC).

Exclusion criteria encompassed non-urothelial histologies such as sarcoma, neuroendocrine tumors, or bladder adenocarcinoma, as well as patients who did not undergo radical cystectomy following chemotherapy.

### 2.2. Exposure

Epidemiological data were collected both before and after neoadjuvant chemotherapy, including prior history of invasive disease, smoking status, and Body Mass Index (BMI).

Biological parameters were recorded at two time points -before chemotherapy (BC) and before surgery (BS)- and included hemoglobin, neutrophil-to-lymphocyte ratio (NLR), creatinine, albumin, and C-reactive protein (CRP).

Data on chemotherapy included the total number of cycles administered, completion status, and adverse events graded according to the Common Terminology Criteria for Adverse Events (CTACAE v5.0). Surgical parameters collected comprised operative duration, intensive care unit stay, and total length of hospitalization.

Postoperative complications were classified using the Clavien-Dindo system and recorded on days 2, 7, and 30, as well as within three months in cases of rehospitalization.

We reported histopathological data included the presence of lymphovascular invasion, ypTNM stage, and the use of adjuvant chemotherapy.

### 2.3. Segmentation

**Figure 1:**
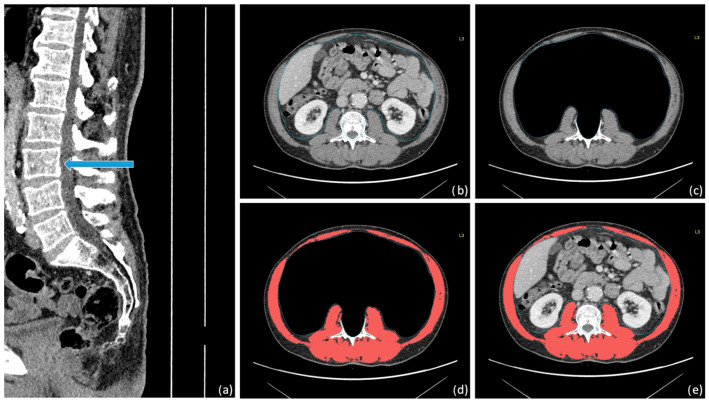
(**a**) CT scan at the L3 vertebra level (blue arrow); (**b**) segmentation of visceral fat and organs; (**c**) removal of visceral fat and organs; (**d**) semi-automatic muscle segmentation of the SMI at the L3 level (red); (**e**) summary view of all regions of interest.

Sarcopenia was evaluated in each patient using baseline CT scans performed BC and BS. When both pedicles were visible, a muscle density threshold from −29 to +150 Hounsfield Units (HU) at the L3 vertebra level was applied. The skeletal muscle area (SMA) was defined as the cross-sectional area of muscle at the L3 level. SMA value was then normalized to the square of the patient’s height to calculate the Skeletal Muscle Index (SMI) (Figure 1).

Two widely used SMI thresholds are used were applied. According to Martin et al. [13], thresholds are BMI- and sex-specific: <43 cm/m^2^ for men with BMI < 25 kg/m^2^, <53 cm/m^2^ for men with BMI ≥ 25 kg/m^2^, and <41 cm/m^2^ for women. According to Fearon et al. [14], thresholds are sex-specific only: <39 cm/m^2^ for women and <55 cm/m^2^ for men, regardless of BMI.

All measurements were independently performed by two blinded experts using OsiriX 14.0 (Pixmeo, Geneva, Switzerland) medical imaging software.

### 2.4. Outcomes

The primary endpoint was overall survival (OS) following neoadjuvant chemotherapy and radical cystectomy, according to the presence or absence of sarcopenia, as defined on pre-chemotherapy CT scans using the Martin and Fearon criteria. Secondary endpoints included progression-free survival (PFS), ypT0N0 rate (pathological complete response (pCR)), Clavien-Dindo-classified complication rates, length of hospital stay, duration of intensive care unit stay, and disease recurrence.

### 2.5. Statistical Analysis

Statistical analyses were conducted using R and RStudio 4.4.0 (R Core Team and Rstudio Team, 2022, Boston, MA, USA). A two-sided significance level of 5% was applied. Categorial variables were summarized as counts and percentage, while continuous variables were described using means and standard deviations, medians, and first and third quartiles. Normally distributed continuous variables were compared using Student’s *t*-test. Otherwise, the Wilcoxon Mann–Whitney test was applied. Categorical variables were compared using the chi-squared test. Survival analyses were performed using the non-parametric Kaplan–Meier method from the date of surgery, and survival curves compared using the log-rank test. Predictive factors were evaluated using univariate and multivariate Cox proportional hazards models. Bivariate analyses of patients’ clinical characteristics were conducted to identify covariates associated with PFS and OS. Variables with a *p*-value < 0.1 in univariate analysis were included in the multivariate Cox model. Results were reported as adjusted hazard ratios (HR) with 95% confidence intervals (CI). SMI BC was used to assess the predictive value and determine the optimal sarcopenia thresholds that maximize the value of the hazard ratio.

## 3. Results

A total of 76 patients received neoadjuvant chemotherapy and underwent cystectomy during the study period. Two patients were excluded from the analysis due to the unavailability of BC CT scans, resulting in a final cohort of 74 patients, whose characteristics are summarized in Table 1.

Regarding chemotherapy, 71.7% of patients received an MVAC-based regimen and 27% received GC-based treatment (one patient received both regimens due to circumstances related to the COVID-19 pandemic). Overall, 83.8% (*n* = 62) of patients completed the full chemotherapy (at least four courses). Grade ≥ 3 adverse events according to CTACAE occurred in 26.0% (n = 19) of patients. Low complications (grade 1 and 2) occurred in 28.8% (*n* = 21), while 45.2% (*n* = 33) experienced no complications during chemotherapy.

Most patients underwent a transileal ureterostomy using the Bricker technique (78.4%). The 90-day rehospitalization rate was 27.4% (*n* = 20). Pathological evaluation showed that 39.2% (*n* = 29) of specimens achieved a pCR and 51.4% (*n* = 38) demonstrated a response defined as stage <ypT2N0M0. Complications of Clavien-Dindo grade ≥3 occurred in 24.3% (*n* = 18) of patients. Low complications (grade 1 and 2) occurred in 54.6% (*n* = 41), while 20.0% (*n* = 15) experienced no complications after surgery.

The median follow-up time was 32.3 months (interquartile (IQ) 18.0–53.5). Disease recurrence occurred in 35.1% (*n* = 26) of patients, predominantly as distant metastases. The median PFS was 25.3 months (IQ 10.8–53.5), and 36.5% (*n* = 27) of patients died during the follow-up period.

### 3.1. Sarcopenia

The median interval between the BC CT scans and the start of chemotherapy was 27.0 days (IQ 15.5–42.0). The median interval between the BS CT scans and surgery was 31.0 days (IQ 21.0–47.75).

Median SMI BC for the overall population was 52.03 cm^2^/m^2^ (IQ 48.08–57.6), with 44.28 cm^2^/m^2^ in women and 53.74 cm^2^/m^2^ in men. Median SMI BS was 49.7 cm^2^/m^2^ (IQ 31.13–54.64), corresponding to a median decrease of 2.94 cm^2^/m^2^. Based on Martin et al.’s criteria, sarcopenia was present in 27% of patients BC and 36.5% of patients BS. Using Fearon et al.’s criteria, sarcopenia was observed in 55.4% of patients BC and 62.3% of patients BS.

We evaluated the predictive value of SMI in our cohort, focusing on sarcopenia BC that maximized the risk of death. For OS, optimal thresholds were identified at 56 cm^2^/m^2^ for men and 39 cm^2^/m^2^ for women. For PFS, the corresponding cut-offs were 52 cm^2^/m^2^ for men and 39 cm^2^/m^2^ for women.

To assess external validity, a blinded review of SMA BC was performed by a second expert in a subset of 39 patients (representing half of the cohort to ensure representativeness). A non-parametric Spearman correlation analysis demonstrated an excellent reproducibility with a correlation coefficient of 0.99 (*p* < 0.001).

### 3.2. Overall Survival

**Figure 2:**
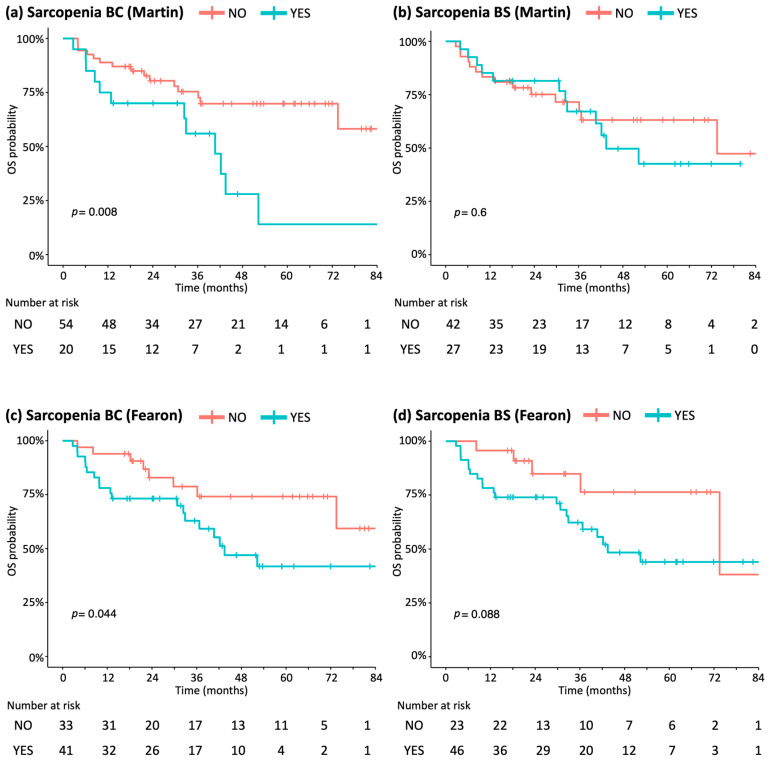
Comparison survival curves according to selected OS values: log-rank analysis. (**a**) According to Martin et al. thresholds BC. (**b**) According to Martin et al. thresholds BS. (**c**) According to Fearon et al. thresholds BC. (**d**) According to Fearon et al. thresholds BS.

Regarding the primary endpoint, univariate analysis (Table 2) showed that sarcopenia BC was significantly associated with worse OS according to Martin’s criteria (HR 2.71; 95% CI [1.26–5.82]; *p* = 0.011). There is a trend toward significance according to Fearon’s criteria (HR 2.3; 95% CI [1.00–5.27]; *p* = 0.05). Comparison of survival curves by the log-rank test confirmed reduced OS in patients with sarcopenia BC for both criteria (Figure 2).

Factors associated with worse OS (Table 2) included older age at surgery (HR 1.05; 95% CI 1.00–1.11; *p* = 0.041), high NLR BC (HR 1.09; 95% CI [1.00–1.18]; *p* = 0.047), high NLR BS (HR 1.39; 95% CI [1.11–1.76]; *p* = 0.005), high ypTNM stage (HR 1.1; 95% CI [1.05–1.15]; *p* < 0.001), and the presence of lymphovascular invasion (HR 5.66; 95% CI [2.48–13]; *p* < 0.001). A high BC hemoglobin appeared to have a protective effect (HR 0.77; 95% CI [0.62–0.95]; *p* = 0.015).

No association were observed between OS and weight or BMI, ASA score, type of urinary diversion, length of hospital stays, postoperative complications, or 90-day rehospitalization. Furthermore, neither the treatment center nor the chemotherapy regimen had a significant effect on OS.

In multivariate analysis, sarcopenia BC (Table 3) remained an independent predictor of poor OS, regardless of the criteria used: Martin’s (HR 3.38; 95% CI [1.25–9.12]; *p* = 0.016) or Fearon’s criteria (HR 4.03; 95% CI [1.13–14.3]; *p* = 0.031). A similar effect was observed for sarcopenia BS (Table 3) with Martin’s criteria (HR 3.7; 95% CI [1.12–12.2]; *p* = 0.032) and Fearon’s criteria (HR 6.08; 95% CI [1.48–24.9]; *p* = 0.012). Other independent factors of poor OS included lower hemoglobin BC, high NLR BC, advanced age at surgery, and the presence of lymphovascular invasion.

### 3.3. Progression-Free Survival

The results of the univariate analysis are presented in Supplementary Tables S1 and S2.

In multivariate analysis, sarcopenia BC was an independent prognostic factor for poor PFS regardless of the criteria used: Martins’s (HR 9.49; 95% CI [2.58–34.9]; *p* < 0.001); Fearon’s (HR 8.39; 95% CI [1.8–39.1]; *p* = 0.007). A similar effect was observed for sarcopenia BS with Martin’s criteria (HR 7.84; 95% CI [1.78–34.5]; *p* = 0.006) and Fearon’s criteria (HR 7.12; 95% CI [1.8–28.2]; *p* = 0.005).

Other independent predictors of poor PFS included a high NLR BC and the presence of lymphovascular invasion.

### 3.4. Secondary Outcomes

In univariate analysis, sarcopenia BC according to Martin’s criteria was significantly associated with higher ypTNM stage (*p* = 0.025), absence of pCR (*p* = 0.003), and lack of chemotherapy response rate (*p* = 0.019). No such associations were observed using Fearon’s criteria of for sarcopenia BS.

Sarcopenia, regardless of the definition, was not significantly associated with prolonged total hospital stay, intensive care unit stay, operative time, 90-day rehospitalization or overall postoperative complications. The only significant association identified was between sarcopenia BS according to Fearon’s criteria and severe complications (Clavien-Dindo ≥ 2) (*p* = 0.021).

## 4. Discussion

Our results confirm the role of pre-chemotherapy and preoperative sarcopenia as independent prognostic factors for OS and PFS in patients with invasive bladder cancer according to both Martin’s and Fearon’s criteria. We derived optimized threshold values for our cohort which closely approximated Fearon’s cut-offs, likely because BMI is not incorporated in that definition. Given the proximity of these newly proposed threshold values to the validated definition, we decided not to conduct additional analyses based on these cut-offs. The observed HRs indicate that sarcopenia confers a substantially increased risk, at least 3-fold. High NLR and low hemoglobin were also associated with survival. However, we did not find the ypTNM stage to be surprising.

Our findings are consistent with the existing literature, although most previous studies involved cohorts of heterogeneous size and used varying diagnostic criteria. Smith et al., in a cohort of 200 patients [15] did not find a significant association between sarcopenia and 30-day complication rates in women. In contrast, other studies have reported significant associations between sarcopenia and prolonged hospital stay [16], as well as poorer overall survival and progression-free survival [17].

Most studies assessing SMI at the L3 vertebral level have applied Martins’ criteria and consistently identified sarcopenia as a negative prognostic factor. Mayr et al. reported a significant association between sarcopenia and a higher incidence of major complications [18]. This adverse effect on outcome was further confirmed in a cohort of 500 patients, showing increased all-cause and disease-specific mortality (HR 1.43 and 1.42, respectively) [19]. Sarcopenia has also been recognized as a risk factor for post-operative venous thromboembolism [20]. Similar associations have been observed using Fearon’s criteria in large cohorts [21].

Few studies have specifically evaluated sarcopenia following neoadjuvant chemotherapy in patients with bladder cancer.

Most previous studies did not find an association between sarcopenia BC and mortality. However, some studies have reported links between sarcopenia and postoperative or post-chemotherapy complications, particularly renal dysfunction [22,23]. These studies were limited by heterogeneous cohorts and varying definitions of sarcopenia.

In contrast, our study demonstrated a strong association between sarcopenia and poor survival by testing multiple thresholds, consistent with the broader literature on sarcopenia in cancer. The lack of significant association with complications in our cohort may be explained by the exclusion of patients who did not undergo cystectomy after neoadjuvant chemotherapy. It is likely that these individuals were excluded due to clinical deterioration or early radiologic progression. Consequently, our study population probably represents the least sarcopenic subset of all patients with bladder cancer treated with chemotherapy. Consequently, patients in our cohort exhibited overall similar rates of completed chemotherapy, regardless of sarcopenia status, contrary to what might have been expected. Similarly, sarcopenia was not associated with postoperative complication rates. This finding may be partly explained by the exclusion of the most fragile sarcopenic patients, who did not undergo cystectomy or did not receive chemotherapy. In addition, less complex surgical techniques were more frequently selected in sarcopenic patients, with a higher proportion of ileal conduit (Bricker) diversions and ureterostomies, which may have reduced postoperative morbidity. Finally, although sarcopenia can be expected to worsen during chemotherapy, we lacked comprehensive data regarding individual longitudinal changes in muscle mass and potential nutritional interventions implemented prior to surgery.

In comparison with previous studies [24,25], we observed a lower response to chemotherapy in patients with sarcopenia defined according to Martin’s criteria. Both chemotherapy response and pathological complete response are well-established prognostic factors in bladder cancer. Nevertheless, as previously mentioned, these patients overall completed neoadjuvant chemotherapy, with rates of complications during chemotherapy or after surgery that were not significantly different from those observed in the other patients. The fact that these patients nonetheless exhibited poorer survival may point toward a difference in tumor response to oncological treatment. This issue remains incompletely understood and warrants further investigation.

In addition to CT-based assessments, several biological markers are recognized as prognostic factors, and these were included in our study. Aside from hemoglobin and NLR, we were unable to identify other significant predictors, likely due to missing data in patient records.

Preoperative anemia has previously been identified as a poor prognostic factor, probably because low hemoglobin limits physical activity [26]. In contrast, albumin reflects both nutritional and inflammatory status. Multiple studies have shown that hypoalbuminemia, generally defined as <35 g/L, is associated with a 5-fold increased risk of complications [27]. In a cohort of over 1000 patients, this association was confirmed linking lower albumin levels to higher rates of 90-day complications and 90-day mortality [28]. Moreover, for each 1 g/L increase in albumin, the risk of complications and 90-day mortality decreased (OR 0.61 and 0.33, respectively). Elevated creatinine is another marker of poor prognosis as it may result from urinary tract obstruction and is often associated with hydronephrosis, a recognized adverse factor [26,29]. NLR has been linked to poorer survival [29], with a cutoff value around >2.5. Higher NLR correlates with more extravesical and lymph node involvement, disease-specific mortality, and all-cause mortality, reflecting both inflammation drive by neutrophils and impaired lymphocyte-mediated antitumor response.

This study has specific biases, notably its retrospective nature and the lack of standardization of delays between CT scans and surgery. Our cohort was of limited size. Although its characteristics appear consistent with those reported in epidemiological studies, our findings require confirmation in larger, adequately powered cohorts. The optimal method for radiologically assessing sarcopenia remains uncertain. Myosteatosis, i.e., the infiltration of fat between and within muscle fibers, may bias CT-based measurements of sarcopenia, and it was not accounted for in this study. Standardized measurement software and widely validated threshold values are needed to improve the accuracy of sarcopenia assessment.

Sarcopenia is a simple and accessible biomarker. The development of artificial intelligence-based tools for radiological assessment offers the potential for automated measurements with near-instantaneous results. We have previously reported on a model using artificial intelligence to measure sarcopenia, which has demonstrated functionality [30]. Sarcopenia is also a reliable indicator of a patient’s overall condition prior to treatment, reflecting both muscular and nutritional status, as well as inflammatory status. Indeed, studies of systemic immune-inflammation have shown associations between BCG response and progression-free survival in patients with localized-disease [31]. Therefore, from the outset of managing invasive carcinoma, urologists could consider corrective and preventive strategies to potentially improve postoperative survival. Identifying sarcopenia at the time of diagnosis may allow the early implementation of tailored muscle-preserving or muscle-strengthening interventions, including nutritional support or referral for specialized nutritional care. If the use of our automated sarcopenia diagnostic tool is validated, this information could be readily obtained from each routinely performed CT scan. Sarcopenia could then be systematically integrated into patient management, alongside established assessments of frailty, such as the G8 screening tool or the Clinical Frailty Scale. We hope to be able to confirm the adoption of these measures in our future practice soon. The methods for correcting sarcopenia have not yet been fully standardized, and future research could propose comprehensive management strategies, including nomograms and tailored corrective interventions for each oncological condition.

## 5. Conclusions

This study evaluated and confirmed the association between sarcopenia and survival in patients with invasive urothelial carcinoma. Sarcopenia was associated with a significantly increased risk of death prior to chemotherapy and surgery. Incorporating biological markers from the standard workup that correlate with sarcopenia could help identify at-risk patients, allowing for early and tailored management.

## Figures and Tables

**Table 1:** Characteristics of patients according to sarcopenia before chemotherapy status using two definitions (Martin and Fearon).

		Martin		Fearon	
Variables	Total Case, *n* (%)	Sarcopenia BC	No Sarcopenia BC	Sarcopenia BC	No Sarcopenia BC
Total case, *n*	74	20	54	41	33
Sex, *n* (%)					
Men	61 (82.4)	18 (90)	43 (80)	39 (95.1)	22 (6.1)
Women	13 (17.6)	2 (10)	11 (20)	2 (4.9)	11 (33.3)
Age at surgery, mean (min–max)	64.5 (38–80)	65.8	62.9	64.6	62.5
Smoker, *n* (%)	64 (86.5)	18 (90)	46 (85.2)	38 (92.7)	26 (78.8)
BMI, median (IQR)					
BC	26.7 (23.8–29.7)	26.7 (24.7–29.5)	26.6 (23.6–29.7)	25.1 (21.7–27.8)	28.2 (25.5–30.7)
BS	25.1 (22.7–28.6)	24.6 (23.0–27.2)	25.5 (22.4–28.9)	23.7 (21.9–25.9)	28.1 (23.5–30.3)
NAC agent, *n* (%)					
GC	20 (27)	6 (30)	14 (25.9)	12 (29.3)	8 (24.2)
MVAC	53 (71.6)	14 (70)	39 (72.2)	29 (70.7)	24 (72.7)
Both	1 (1.4)	0	1 (1.9)	0	1 (3)
Complete chemotherapy, *n* (%)	62 (83.8)	16 (80)	46 (85.1)	34 (83)	28 (84.8)
Complication grade, *n* (%)					
1	1 (1.4)	0	1 (1.9)	1 (2.4)	0
2	20 (27.4)	7 (35)	13 (24.1)	12 (29.3)	8 (24.2)
3	16 (21.9)	3 (15)	13 (24.1)	8 (19.5)	8 (24.2)
4	3 (4.1)	1 (5)	2 (3.7)	1 (2.4)	2 (6)
ASA score, *n* (%)					
1	3 (5.1)	0	3 (5.6)	3 (7.3)	0
2	32 (5.2)	7 (35)	25 (46.3)	15 (36.6)	17 (51.2)
3	24 (40.7)	8 (40)	16 (29.6)	13 (31.7)	11 (33.3)
Diversion type, *n* (%)					
Ureterostomy	1 (1.4)	1 (5)	0	1 (2.4)	0
Bricker procedure	58 (78.4)	17 (85)	41 (76)	34 (83)	24 (72.7)
Continent type	15 (20.3)	2 (10)	13 (24.1)	6 (14.6)	9 (27.3)
ypTNM, *n* (%)					
T0N0M0	29 (39.2)	3 (15)	26 (48.1)	12 (29.3)	17 (51.2)
N+	8 (10.8)	2 (10)	6 (11.1)	5 (12.2)	3 (9.1)
Positive surgical margin, *n* (%)	3 (4.1)	2 (10)	1 (1.9)	2 (4.9)	1 (3)

**Table 2:** Factors associated with OS in univariate analysis.

				BC			BS		
	HR	95% CI	*p*	HR	95% CI	*p*	HR	95% CI	*p*
Sarcopenia									
Martin et al.				2.71	1.26–5.82	0.011	1.23	0.57–2.68	0.6
Fearon et al.				2.3	1.00–5.27	0.05	2.28	0.86–6.06	0.1
Age at surgery	1.05	1.00–1.11	0.041						
High weight				1.01	0.98–1.03	0.5	1.00	0.98–1.02	>0.9
High BMI				1.02	0.94–1.11	0.6	0.98	0.91–1.08	0.9
High creatinine				1.01	0.99–1.04	0.2	1.00	0.99–1.00	0.5
High albumin				0.96	0.86–1.07	0.5	1.03	0.98–1.08	0.3
High hemoglobin				0.77	0.62–0.95	0.015	0.94	0.73–1.21	0.6
High CRP				1.00	0.98–1.03	0.7	1.01	0.95–1.08	0.7
High NLR				1.09	1.00–1.18	0.047	1.39	1.11–1.76	0.005
High ypTNM	1.1	1.05–1.15	<0.001						
Complete chemotherapy	0.54	0.22–1.34	0.2						
Operating duration	0.82	0.64–1.05	0.11						
Lymphovascular invasion	5.66	2.48–13	<0.001						

**Table 3:** Factors associated with overall survival, considering sarcopenia BC and BS: Cox analysis.

	Martin			Fearon		
	HR	95% CI	*p*	HR	95% CI	*p*
			**BC**			
Sarcopenia	3.38	1.25–9.12	0.016	4.03	1.13–14.3	0.031
High hemoglobin BC	0.56	0.36–0.86	0.009	0.64	0.43–0.95	0.025
High NLR BC	0.81	0.7–0.94	0.006	0.8	0.68–0.94	0.006
High ypTNM	1.00	0.93–1.08	>0.9	1.02	0.95–1.11	0.6
Lymphovascular invasion	8.7	1.87–40.4	0.006	12.8	2.65–61.6	0.002
			**BS**			
Sarcopenia	3.7	1.12–12.2	0.032	6.08	1.48–24.9	0.012
High hemoglobin BS	1.51	0.96–2.39	0.077	1.16	0.7–1.92	0.6
High NLR BS	1.62	1.06–2.49	0.026	1.42	0.99–2.04	0.059
High ypTNM	0.96	0.87–1.06	0.4	1.00	0.91–1.1	>0.9
Lymphovascular invasion	18.9	2.92–122	0.002	9.13	1.8–46.2	0.008

## Data Availability

The datasets presented in this article are not readily available because the data are part of an ongoing study.

## Supplementary Materials

The following supporting information can be downloaded at: https://www.mdpi.com/article/10.3390/cancers18020222/s1, Supplementary Table S1. Association between sarcopenia and PFS: univariate analysis. Supplementary Table S2. Factors associated with PFS: univariate analysis.
