# Supplementary material for: Sarcopenia Predicts Mortality in Bladder Cancer with Neoadjuvant Chemotherapy: A Multicenter Study
Source: Cancers (Basel). 2026 Jan 11;18(2):222. doi: 10.3390/cancers18020222 (PMC12838778; doi:10.3390/cancers18020222)
Supplement: Supplementary file 1 [file cancers-18-00222-s001.zip › Table S1.pdf]

	**Sarcopenia BC**			**Sarcopenia BS**		
	HR	95% CI	p	HR	95% CI	p
**Martin**	2.38	1.07-5.29	0.033	1.13	0.51-2.52	0.8
**Fearon**	1.63	0.74-3.61	0.2	1.07	0.47-2.42	0.9
