# Supplementary material for: Sarcopenia Predicts Mortality in Bladder Cancer with Neoadjuvant Chemotherapy: A Multicenter Study
Source: Cancers (Basel). 2026 Jan 11;18(2):222. doi: 10.3390/cancers18020222 (PMC12838778; doi:10.3390/cancers18020222)
Supplement: Supplementary file 1 [file cancers-18-00222-s001.zip › Table S2.pdf]

				BC			BS		
	HR	95% CI	p	HR	95% CI	p	HR	95% CI	p
**SMI**				1.01	0.97-1.05	0.5	1.00	0.96-1.05	>0.9
**Difference between SMI BC and BS**	1.00	0.89-1.13	>0.9						
**Age at surgery**	1.04	0.99-1.09	0.1						
**Weight**				1.01	0.99-1.03	0.4	1.01	0.98-1.03	0.5
**BMI**				1.05	0.98-1.14	0.2	1.04	0.96-1.13	0.3
**Creatinemia**				1.02	1.0-1.04	0.14	1.00	0.99-1.00	0.5
**Albumin**				0.96	0.87-1.06	0.4	1.05	1.00-1.10	0.073
**Hemoglobin**				0.83	0.68-1.02	0.079	**0.71**	**0.53-0.95**	**0.021**
**CRP**				1.02	0.99-1.05	0.2	1.06	0.98-1.14	0.13
**NLR**				**1.11**	**1.01-1.21**	**0.022**	**1.3**	**1.01-1.67**	**0.044**
**ypTNM**	**1.12**	**1.07-1.17**	**<0.001**						
**Complete chemotherapy**	0.7	0.26-1.87	0.5						
**Operating duration**	0.91	0.71-1.16	0.4						
**Lymphovascular invasion**	**7.44**	**3.25-17.1**	**<0.001**						
